# Evaluating the glymphatic system via magnetic resonance diffusion tensor imaging along the perivascular spaces in brain tumor patients

**DOI:** 10.1007/s11604-024-01602-7

**Published:** 2024-05-31

**Authors:** Gabriela Villacis, Aileen Schmidt, Justus C. Rudolf, Hannes Schwenke, Jan Küchler, Peter Schramm, Patricia Ulloa

**Affiliations:** 1 Department of Neuroradiology, University Hospital Schleswig-Holstein (UKSH), Luebeck, Germany; 2 Department of Neurosurgery, University Hospital Schleswig-Holstein (UKSH), Luebeck, Germany

**Keywords:** Glymphatic system, Neurofluids, DTI-ALPS (diffusion tensor imaging along perivascular spaces), Brain tumor

## Abstract

**Purpose:**

To investigate glymphatic system function in patients with brain tumors, including both primary and secondary tumors, using diffusion tensor imaging along perivascular spaces (DTI-ALPS).

**Methods:**

We retrospectively analyzed the MR DTI of 24 patients with unilateral brain tumors and compared them with age and sex-matched controls. We compared the DTI-ALPS index of the ipsi- and contralateral brain hemispheres. The region of interest was placed in the periventricular vessels adjacent to the lateral ventricles. Differences between sex, age, and kind of tumor (primary or brain metastasis) were evaluated. Correlations between DTI-ALPS index and age and the tumor's apparent diffusion coefficient (ADC) were also investigated.

**Results:**

The DTI-ALPS index was significantly lower (*p* < 0.05) in the tumor-affected hemisphere (mean = 1.26 ± 0.24) than contralateral (mean = 1.43 ± 0.28). A comparison with healthy controls revealed no significant difference on the matched ipsilateral side. However, the DTI-ALPS index of the contralateral side of the patients was larger than the HC. Additionally, no statistically significant differences were found when analyzing the DTI-ALPS index vs. age, sex, and tumor entity. Additionally, we did not find a correlation between the DTI-ALPS index and patient age or tumor ADC.

**Conclusion:**

The decreased DTI-ALPS index in the tumor-affected hemisphere may be related to impaired glymphatic system function. However, cancer is often a systemic disease; thus, the DTI-ALPS index from the contralateral brain hemisphere may not generally be considered as a normal control. Nonetheless, the DTI-ALPS index does not only reflect diffusion in the perivascular spaces but it can also be influenced by factors such as axonal degeneration. Therefore, it does not directly reflect brain waste clearance and changes in the index should be interpreted carefully.

## Introduction

The glymphatic system (GS) was recently proposed to be the waste clearance system of the brain [1, 2], reports describing how the cerebrospinal fluid (CSF) flows into the interstitial space and then to the venous perivascular space to continue into the lymphatic vessels, removing metabolic waste products from the brain. This is possible due to the arterial pulsation in the brain, which creates waves, pushing the fluid along the tissue [2], and as a result of pressure differences due to respiration [3, 4] and the production of CSF in the choroid plexus [4]. Owing to this flow of CSF and interstitial fluid (ISF) in the perivascular space, metabolic waste is eliminated from the interstitial space, and other substances such as glucose, neurotransmitters and amino acids are distributed there.

In a healthy GS, metabolites are removed from the interstitial space via aquaporin-4 water channels. These are found on the astrocytic feet and facilitate the flow of CSF in the neuropil. However, some diseases or inflammatory processes can cause GS dysfunction, leading to the pathological accumulation of harmful metabolites, including amyloid ß and tau proteins [1, 5, 6], which are usually related to neurodegeneration. Additionally, failure in the GS function can also affect the cognition of patients [7], and cause edema in stroke [8] or in tumors [9–11]. The GS has also been observed to be more active during sleep [12] because then resistance is lower in the tissue, facilitating the flow of CSF, which serves to remove metabolites. This process has been suggested as a means of regenerating the brain during sleep.

Diffusion tensor imaging along perivascular spaces (DTI-ALPS) [13] is a promising noninvasive technique to evaluate GS function. The DTI-ALPS index is a diffusivity ratio that represents water diffusion along the perivascular spaces of the medullary veins. Decreases in the DTI-ALPS index have been reported in several neurodegenerative diseases. This index has been used in many studies as a measure of GS function, such as in Parkinson´s disease [14–21], Alzheimer´s disease [5, 13, 22, 23], stroke [8], and epilepsy [24–27], where a lower DTI-ALPS index may be associated with impaired glymphatic function [1, 28]. Importantly, in hydrocephalus patients, this index has shown to be significantly reduced, pointing to glymphatic system dysfunction, as demonstrated by delayed clearance of intrathecally administrated gadobutrol [29, 30].

Therefore, the DTI-ALPS index appears to represent a new biomarker for GS function. To our knowledge, GS function has been studied in patients with brain tumors such as gliomas [9], meningiomas [10], and brain metastases [11] using DTI-ALPS. However, only the research in meningiomas consider the DTI-ALPS index of the contralateral side.

The study of both hemispheres is interesting because e.g., in patients with epilepsy [25, 27, 31, 32], the DTI-ALPS index on the ipsilateral side was lower than on the contralateral side, suggesting more severe glymphatic system function in the hemisphere ipsilateral to the epileptogenic foci. Other examples include ischemic [8, 33] and hemorrhagic [34] stroke and traumatic brain injury[35], where the ipsilateral side had a reduced DTI-ALPS index in comparison with the contralateral hemisphere. Additionally, considering that in normal subjects, there have been inconsistent results in whether there are significant differences between dominant and non-dominant brain hemispheres, the lateralization of the DTI-ALPS index in HC and patients requires further research.

Therefore, we analyzed GS function in both primary and secondary tumors in the brain using DTI-ALPS in the ipsi- and contralateral sides of the brain, and made a comparison with healthy controls (HC). We hypothesized that DTI-ALPS index would be lower on the side of the brain with the tumor than on the contralateral side, which might be linked to brain waste clearance dysfunction. We also evaluated differences in DTI-ALPS index between sex, age, and tumor histology (primary vs. brain metastasis).

Additionally, as the peritumoral edema of cerebral metastases has been reported as positively correlated with tumor ADC and inversely with the DTI-ALPS index [11] we investigated if there is a correlation between the tumors' ADC and DTI-ALPS in our cohort.

## Methods

### Clinical information

The patients’ clinical history and MR images were retrospectively inspected to collect information regarding age, sex, and kind of tumor. To identify statistically significant differences, the study included 30 consecutive patients who had preoperative functional MRI and DTI between September 2020 and October 2022 and was approved by the ethics committee of the University of Luebeck. To be part of this study, the patients needed to be older than 18 years, and the diffusion images needed to be free of movement artifacts, and the area of the periventricular veins should not be affected by the tumor (six patients were excluded). This resulted in a total of 24 patients analyzed in this study. The patients were informed about the possible risks associated with an MRI examination as part of clinical routine and they signed a general consent form. Detailed demographic information is shown in Table 1. For comparison, we included healthy participants as a control to match the patients for sex and age (within ± 2 years) with normal brain MR examination and without any history of neurological disorders.

**Table 1:** Demographic information

Patient nr	Age	Sex	DTI-ALPS ipsilateral	DTI-ALPS contralateral	Tumor’s ADC mm^2^ s^−1^	Event	Type of tumor	Details	Tumor location	PTBE
1	24	Male	1.10	1.37	1601	Local recurrence	Primary	Diffuse Astrocytoma	Left frontal	No
2	73	Female	0.98	1.18	525.5	Distance recurrence	Metastasis	B-cell lymphoma	Left high frontal	No
3	71	Female	0.97	0.98	75.5	Distance recurrence	Metastasis	Bronchial carcinoma	Left high frontal	Yes^ROI^
4	68	Female	1.12	1.31	810	Distance recurrence	Metastasis	Mammary carcinoma	Left occipital	Yes
5	48	Male	1.14	1.15	1220	1. diagnosis	Primary	Unclear*	Left temperofronto-parieto insular	Yes^ROI^
	56	Female	excluded		912	Distance recurrence	Metastasis	Mammary carcinoma	intraventricular	Yes
7	38	Male	0.96	1.30	1287	Local recurrence	Primary	Glioblastoma	Left frontal	Yes^ROI^
8	59	Male	excluded		1182	Local recurrence	Primary	Glioblastoma	Left frontal	Yes
9	53	Male	1.67	1.81	1558	1. diagnosis	Primary	Diffuse Astrocytoma	Left temporoinsular	Yes
10	51	Male	1.39	1.51	1333	Local recurrence	Primary	Diffuse Astrocytoma	Right frontotemporoinsular	No
11	51	Male	1.34	1.58	1291	Local recurrence	Primary	Diffuse Astrocytoma	right Frontotemporoinsular	No
12	56	Female	2.03	2.28	1719	1. diagnosis	Primary	Convexity meningioma	Left convexity	No
13	71	Male	1.29	1.30	1845	Local recurrence	Primary	Glioblastoma	Left temporal	Yes
14	72	Female	excluded		2927	Distance recurrence	Metastasis	Endometrial adenocarcinoma	Right frontoparietal	Yes
15	67	Female	1.54	1.44	2688	Distance recurrence	Metastasis	Clear cell renal carcinoma	Left temporal	Yes
16	60	Female	1.13	1.59	2734	Local recurrence	Primary	Multifocal glioblastoma	Left parietooccipital and left occipital	Yes
17	67	Female	1.35	1.35	2883	Distance recurrence	Metastasis	Renal cell carcinoma	Left parietooccipital	Yes
18	43	Male	1.28	1.62	1769	Distance recurrence	Metastasis	Bronchial carcinoma	Left temporal	Yes
19	62	Female	Excluded		824	1. diagnosis	Primary	Glial tumor	Left frontoparietal	Yes
20	70	Male	1.42	1.58	1556	1. diagnosis	Primary	Glioblastoma	Right high frontal	Yes
21	58	Male	1.20	1.22	1140	1. diagnosis	Primary	Glioblastoma	Left temporal	Yes
22	61	Female	1.01	1.19	1798	Local recurrence	Primary	Multifocal glioblastoma	Left parietoocipital and left occipital	Yes
23	57	Male	1.18	1.32	2057	Local recurrence	Primary	Glioblastoma	Left temporoinsular	Yes^ROI^
24	61	Female	Excluded		1012	1. diagnosis	Primary	Glioblastoma	Left temporoparietal	Yes
25	50	Male	1.34	1.55	2257	Local recurrence	Primary	Oligodendroglioma grade III	Left parietal	Yes
26	54	Male	Excluded		711	1. diagnosis	Primary	Glioblastoma	Right frontoparietal	Yes
27	32	Male	1.30	1.40	1576	1. diagnosis	Primary	Oligodendroglioma grade II	Left frontal	Yes
28	58	Female	1.16	1.17	1059.5	Distance recurrence	Metastasis	Bronchial carcinoma	Left parietal and left occipital	Yes
29	59	Female	1.24	1.89	1628	Local recurrence	Primary	Glioblastoma	Left temporoparieto-occipital	Yes^ROI^
30	72	Male	1.03	1.25	2444	1. diagnosis	Primary	Glioblastoma	Left temporal	Yes

*Patient nr. 5 was a referral from other institution, ^ROI^ means that the ROI was placed in the PTBE

### MRI acquisition parameters

**Fig. 1:**
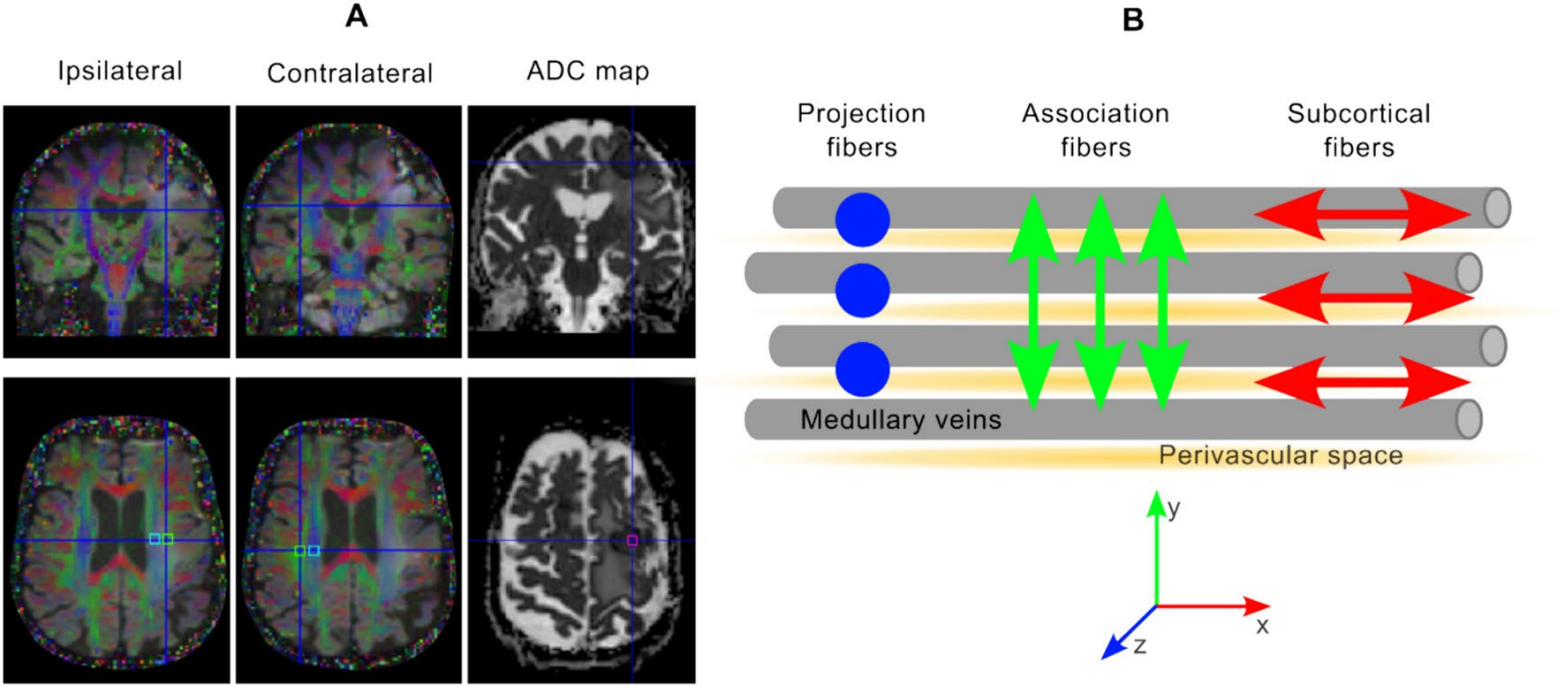
**A** Tumor patient (patient no. 3, female, 71 years) with cerebral metastasis (bronchial carcinoma, left frontal). From left to right: fractional anisotropy maps overlaid on a diffusion-trace image and an ADC map. Squares mark the approximate positions of the ROIs (blue: projection; green: association; and pink: tumor ADC). Note that the ROIs on the ipsilateral side are located inside the peritumoral edema. **B** Concept of DTI-ALPS. The projection area (in blue) (i.e., a region dominated by projection fibers in the acquired slice) and association area (in green) contain axonal fibers in the feet-head and anterior–posterior directions, respectively. The perivascular space runs perpendicular to these fibers. By using DTI, the diffusivity along the x-axis in the two ROIs (DxxProj and DxxAssoc) would partially represent the diffusivity along the perivascular space in the area next to the lateral ventricles. In the projection area, the dominant fibers are along the z-axis. Therefore, Dxx and Dyy are perpendicular diffusivities while in the association area; the main direction of the fibers is along the y-axis (Dxx and Dzz are the perpendicular diffusivities). The DTI-ALPS index is the ratio between the mean of two diffusivities perpendicular to the primary fiber orientation in the projection and association areas

All MRI studies were performed using a 3-T clinical scanner (Magnetom Vida, Siemens Healthineers, Erlangen, Germany) with a 20-channel head coil. The DTI scan was performed using a 2D single-shot echo-planar-imaging (EPI) acquisition, with the following parameters: TE/TR = 92/6100 ms; 64 diffusion directions, 2 b-values = 0 and 1000 s/mm^2^; field-of-view: 200 × 200 mm^2^; matrix size: 100 × 100; slice thickness: 2 mm, and voxel size: 2 × 2 mm^2^. This scan is part of the MR tumor protocol at our clinical center and takes 7 min. The ADC map was calculated using the trace image and b = 0. The tumor ADC was calculated over an ROI placed at the center of the tumor (area: 5.3 mm^2^) (see Fig. 1A).

### Image post processing and analysis

All images were analyzed in MATLAB 2022a (The MathWorks Inc, Natick, Massachusetts, United States) using statistical parameter mapping (SPM) 12 (https://www.fil.ion.ucl.ac.uk/spm) and the diffusion II toolbox (https://sourceforge.net/projects/spmtools/). The diffusion toolbox includes motion correction based on the b = 0 image (with gradient direction correction), and fractional anisotropy, diffusion tensor, and tensor decomposition are calculated. Image quality control was performed by board-certified neuroradiologists.

### Calculation of the DTI-ALPS index

The DTI-ALPS [13] method uses two ROIs positioned on a single slice at the level of the lateral ventricles. In that region, the direction of the parenchymal vessels is approximately perpendicular to the ventricle wall. Therefore, the perivascular space is in the same direction: right-left in the patient coordinate system. This concept is explained in Fig. 1B. The DTI-ALPS index was measured by positioning a cubically shaped region-of-interest (ROI) of 2 × 2 voxels over one 2-mm thick slice (corresponding to an area of 4 × 4 mm^2^). As a tumor affects brain symmetry, it was not possible to mirror the position of the ROIs in both hemispheres. Two neuroradiologists, with 1 and 10 years of experience in dedicated brain tumor imaging, supported the ROI positioning in consensus on the projection and association areas on the ipsi- and contralateral side (see Fig. 1A), avoiding peritumoral brain edema (PTBE) when possible. This was done based on the fractional anisotropy map next to the body of the ventricle.

By using the diagonal elements from the diffusion tensor, the DTI-ALPS index was calculated as [13].

DTI-ALPS index = $\frac{\text{mean }(\text{Dxxproj},\text{ Dxxass})}{\text{mean }(\text{Dyyproj},\text{ Dzzass})}$, where Dxx, Dyy and Dzz are the diagonal elements of the tensor.

### Statistical analysis

MATLAB’s statistical toolbox with a 0.05 significance level was used for statistical analysis. The difference in the DTI-ALPS index between the tumor and contralateral side and the HC matching were assessed using the Wilcoxon signed rank test (signrank in Matlab). DTI-ALPS index differences according to age (< 55 vs. > 55 years), sex (male vs. female), and type of tumor (primary vs. secondary) were evaluated using the Mann–Whitney U-test (ranksum in Matlab). In addition, we investigated the relationship between the DTI-ALPS index and patient ages and tumor ADC using linear regression.

## Results

### Demographics

In this study, 14 male and 10 female patients aged between 24 and 73 years old (mean = 56.58 ± 12.71) were included. Among these patients, 7 had glioblastomas, 4 diffuse astrocytoma, 3 bronchial carcinoma, 2 oligodendroglioma, 2 multifocal glioblastoma, 2 renal cell carcinoma, 1 mammary cell carcinoma, 1 B-cell lymphoma, 1 convexity meningioma, and 1 with an unclear diagnosis. From these patients, 17 had primary tumors (10 patients with local recurrences and 7 with first diagnosis) and 7 cerebral metastases. Five patients did not have PTBE. For more details, see Table 1.

Twelve HC between 24 and 71 years old (8 males, 4 females; mean age = 52.25 ± 14.20 years) were included. Details are shown in Table 2.

**Table 2:** Information of the healthy controls used for matching the patients

			DTI-ALPS index		
Control nr	Sex	Age/year	Right hemi	Left hemi	Matched patient
1	m	33	1.51	1.52	27
2	m	24	1.52	1.31	1
3	f	57	1.38	1.39	12, 16, 28, 29
4	m	59	1.31	1.31	21, 23
5	m	41	1.45	1.47	7
6	m	70	0.90	0.85	13, 20, 30
7	f	67	1.37	1.46	4, 15, 17
8	m	49	1.23	1.14	5
9	f	71	1.42	1.25	2, 3
10	f	61	0.88	0.93	22
11	m	43	1.51	1.42	18
12	m	52	0.99	1.09	9, 10, 11, 25
Mean ± std		52.25 ± 14.20	1.29 ± 0.23	1.26 ± 0.21	
Median (Q1, Q3)		54 (42.50, 62.5)	1.37 (1.17, 1.46)	1.31 (1.13, 1.43)	

### DTI-ALPS index analysis

The DTI-ALPS index calculations and comparisons between the ipsi- and contralateral side of the tumors, age, sex, and tumor type are summarized in Table 3. Due to the position of the tumor and the extent of the PTBE, the ipsilateral ROIs were placed in the area of the edema in 5 patients. However, no significant differences were observed when the ROI was located inside or outside the PTBE (Mann–Whitney U-Test, *p* = 0.065).

**Table 3:** Comparisons of the DTI-ALPS index according to tumor side, age, sex, and type of tumor

		DTI-ALPS index		
Parameter	Participants	Mean ± std	Median (Q1, Q3)	*p*
Hemisphere				6.7e–5*
Tumor ipsilateral	24	1.26 ± 0.24	1.22 (1.12, 1.34)	
Tumor contralateral		1.43 ± 0.28	1.36 (1.24, 1.58)	
Age				0.51
< 55	9	1.28 ± 0.19	1.30 (1.14, 1.34)	
> 55	15	1.24 ± 0.26	1.18 (1.08, 1.32)	
Sex				0.46
Male	14	1.26 ± 0.17	1.29 (1.15, 1.34)	
Female	10	1.25 ± 0.31	1.15 (1.04, 1.32)	
Type of tumor				0.53
Primary tumor	17	1.28 ± 0.25	1.24 (1.13, 1.34)	
Cerebral metastasis	7	1.20 ± 0.19	1.16 (1.05, 1.32)	

A significant difference is marked with *(*p* < 0.05). Indices are presented as mean ± standard deviation and median (1st quantile, 3rd quantile)

**Fig. 2:**
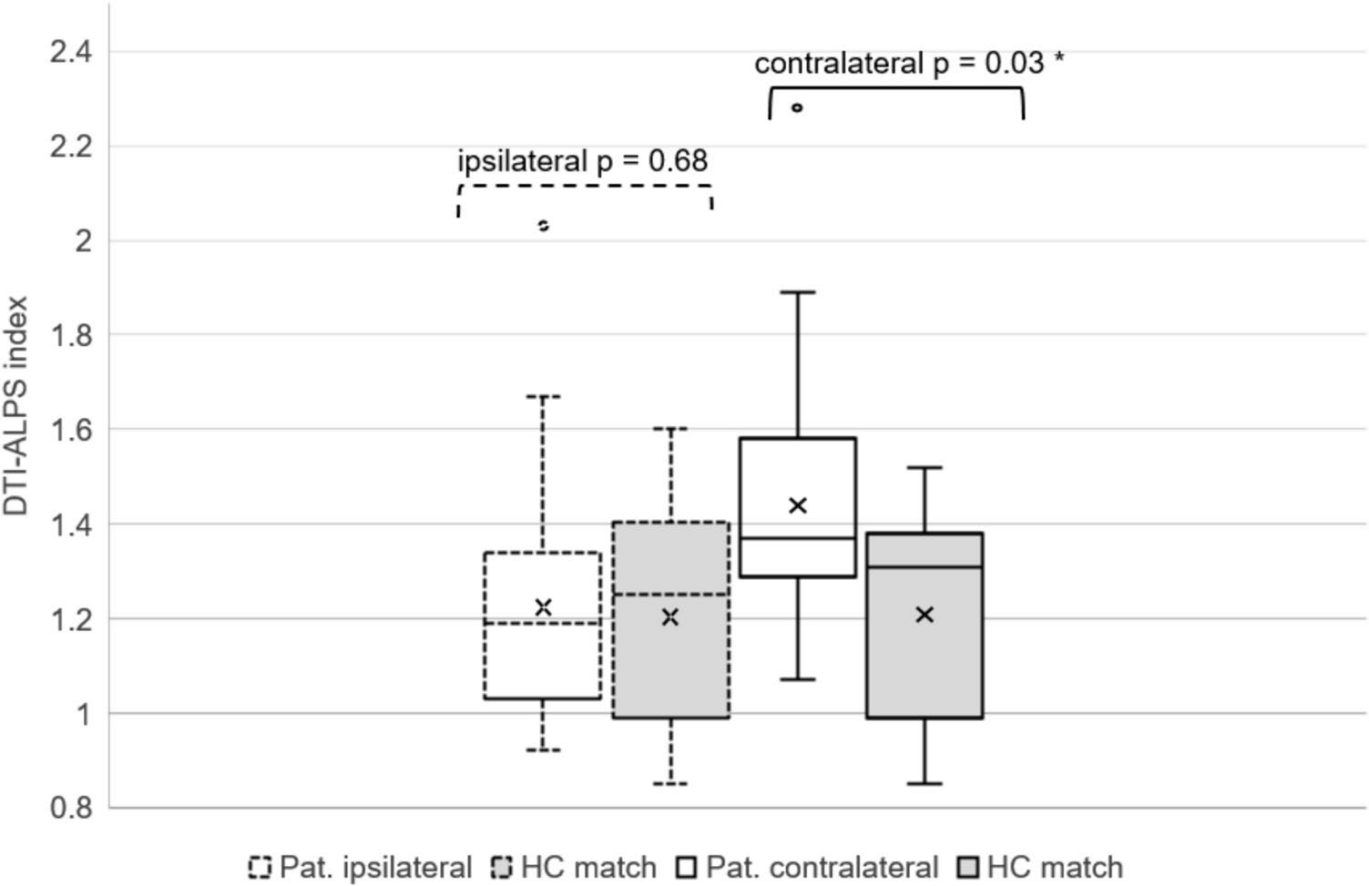
Boxplot showing the difference between the DTI-ALPS index of the tumor side of the brain vs. the contralateral side (*p* < 0.05) and HC. The significant difference is marked with *. For specific values see Table 1 and Table 3

The most relevant finding of this study is that the DTI-ALPS index was significantly lower on the side of the brain with the tumor (DTI-ALPS index = 1.26 ± 0.24) than in the contralateral hemisphere (DTI-ALPS index = 1.43 ± 0.28) (*p* = 6.7e–5) (see Fig. 2). When comparing DTI-ALPS index in patients and controls, we found no significant difference between the DTI-ALPS index between the ipsilateral side of tumor patients and age-sex matched control (HC mean: 1.22 ± 0.22) (*p* = 0.68). However, there is a significant difference (*p* = 0.03) when comparing the patients' contralateral side to HC (HC mean = 1.23 ± 0.22), where the DTI-ALPS index in the patients' contralateral side is larger than the one from the HC. This contradicts the finding of Toh et al. in their study on meningiomas[10]. There is no significant difference between the right and left hemispheres in HC.

**Fig. 3:**
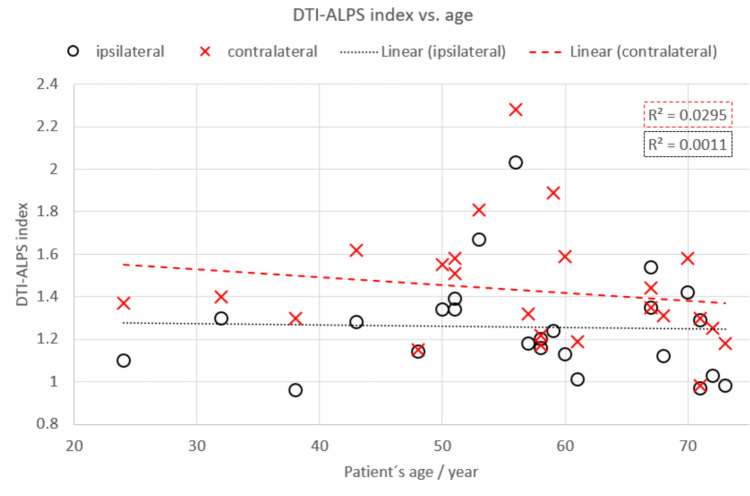
No linear relationship was found between the DTI-ALPS index and patient’s age, per hemisphere

**Fig. 4:**
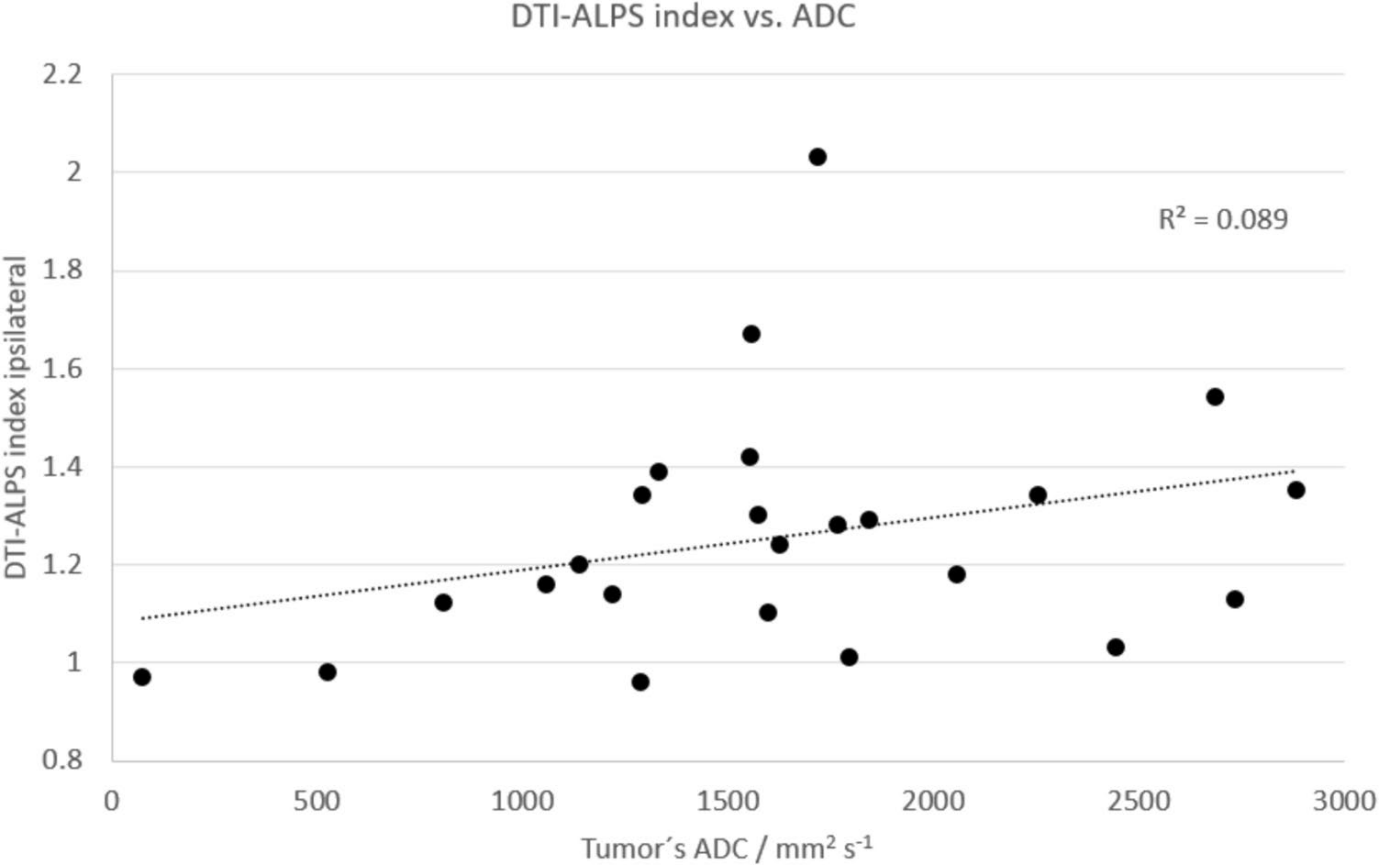
No linear correlation was observed between the DTI-ALPS index and tumor ADC

Additionally, we did not find any differences in the DTI-ALPS index according to age, sex, or type of tumor (*p* > 0.05) (see Table 3). Furthermore, no linear correlation was found between the DTI-ALPS index and patient age (*R*^2^ = 0.0011, *p* = 0.88 and *R*^2^ = 0.003, *p* = 0.422 for the ipsi- and contralateral side, respectively) and tumor ADC (*R*^2^ = 0.09, *p* = 0.16) (see Fig. 3 and 4). The average tumor ADC was 1618.42 ± 665.29 mm^2^s^−1^.

## Discussion

In the present study, we analyzed retrospectively the DTI-ALPS index in patients with brain tumors, including both primary brain tumors and cerebral metastases, and compared them to healthy subjects. We compared the DTI-ALPS index of the tumor-affected hemisphere to that of the contralateral side and found statistically significant differences between the hemispheres. In comparison with HC, we found no difference between the patients' ipsilateral and matched DTI-ALPS index. In addition, the DTI-ALPS index of the contralateral side of the patients was larger than the one from HC, contradicting Toh et al. study on meningiomas [10].

On the other hand, we did not find significant group differences according to age, sex, or type of tumor. Furthermore, we did not find a linear correlation between DTI-ALPS index and age and tumor ADC. To our knowledge, only three publications so far have used the DTI-ALPS method to assess GS function in patients suffering from brain tumors [9–11]. However, only the study in meningioma [10] considered the tumor’s contralateral side and HCs. The DTI-ALPS index in brain tumors has been evaluated by Toh et al. [9–11]. First, they analyzed the formation of PTBE in meningiomas [10]. In that study, they compared the DTI-ALPS index in patients with meningiomas with and without PTBE and HC. They found that the DTI-ALPS index was larger in patients without PTBE than in both healthy subjects and patients with edema. There was no difference between the patients with edema and the HC. These findings disagree with our results, where there is no significant difference between the ipsilateral side and the matched HC and also that the contralateral DTI-ALPS index was larger in tumor patients than HC.

Previous publications have also observed no significant differences between patients and controls, such as in Parkinson's disease [16], focal epilepsy [25] and migraine [36]. However, to our knowledge, no publication has reported a higher index in patients than in healthy subjects. One could hypothesize that the diffusion along perivascular spaces in the contralateral side is abnormally enlarged in patients in an attempt to compensate for the increased amount of brain waste products due to the increased tumor's metabolic activity, tissue compression and general neuroinflammation. However, no definitive conclusions can be made yet and further research is required.

Besides, one needs to consider that Toh et al. [10] mainly studied PTBE in extra-axial tumors and its influence on the DTI-ALPS index, and we did not make a separation between patients with and without PTBE.

Additionally, contrary to our findings, they did not find a significant difference between ipsi- and contralateral hemispheres in meningioma patients, without further discussion. We speculate that for this reason, they did not investigate the DTI-ALPS index in the tumor’s non-affected contralateral side in further research. We could hypothesize that intra- and extra-axial tumors may influence the DTI-ALPS index using different mechanisms. However, we cannot draw definitive conclusions, and more research is needed [10].

Secondly, Toh and Siow [9] investigate the DTI-ALPS index in patients with gliomas in the hemisphere of the tumor. They compared the DTI-ALPS index in patients with different stages of glioma, the type of isocitrate dehydrogenase 1 (IDH1), and the sex of the patients. They found a significant difference in the DTI-ALPS index between patients with a lower glioma grade and those with a higher-grade glioma (II/III grade vs. IV grade glioma). Additionally, the index for the mutant IDH1 was higher than that for the wild-type mutation. They did not include HC in this research. It could have been interesting to compare our work with Toh et al. glioma study [9]. Unfortunately, it lacks ipsi—contralateral comparison and does not include HC. Consequently, we cannot compare our results.

Lastly, they studied perivascular edema in brain metastases [11]. They found that high ADC tumor values and low DTI-ALPS index were related to large edema volumes. This suggests that high volumes of edema may be related to intratumoral water diffusivity, disturbing glymphatic function. We did not find a significant relationship between the tumor's ADC and DTI-ALPS index. However, as the main focus of Toh et al. [11] is PTBE; therefore, making a comparison between our results is not possible.

In our opinion, the contralateral hemisphere should be analyzed in particular when considering gliomas as it must increasingly be assumed that higher-grade gliomas do not only show abnormalities in pathologically contrast-enhancing lesions on MRI. Cerebral glioma must increasingly be evaluated as diseases of larger parts of the brain, if not of the entire brain. Therefore, determining the DTI-ALPS index in the contralateral hemisphere, too, is significant.

As expected, we found that the DTI-ALPS index in the ipsilateral hemisphere was lower than that on the contralateral side. This might suggest impaired glymphatic function on the side of the brain with the tumor. However, the DTI-ALPS index does not solely represent diffusion in the perivascular space, but also other processes such as axonal degeneration [37]; and, therefore, cannot be directly linked to the GS function. Additionally, due to different tumor locations, the anatomy of the brain and periventricular veins may be altered and measuring the diffusivity along the perivascular spaces in the ipsilateral side may be not be totally free from unwanted influences due to anatomical displacements. Therefore, we excluded patients that due to the position of the tumor, the area of the periventricular veins was affected. Additionally, intra-axial tumors can directly disrupt the microstructure of the brain and directly affect the water diffusion in the brain due to e.g. cellular growth or tumor cell infiltration. On the other hand, extra-axial tumors, due to mass effect, can compress and exert pressure on adjacent structures but typically do not directly infiltrate brain tissue. This could suggest the existence of different brain waste clearance mechanisms. Also, as the DTI-ALPS index is measured in the deep white matter, it is not surprising that the apparent diffusion coefficients in the tumor-affected hemisphere will differ from the contralateral side in the case of intra-axial tumors. Nevertheless, both tumor types could compress the brain, or the extra-axial tumors could infiltrate the perivascular spaces, like the periventricular veins. Therefore, extra-axial tumor infiltration into brain tissue might have different influences on deep white matter than non-infiltrated ones, and this might need to be investigated separately.

In our cohort, we did not find significant gender differences regarding the DTI-ALPS index results. This is consistent with the findings of other DTI-ALPS tumor-related studies [9–11]. Previous studies investigating the DTI-ALPS index difference between males and females reached the same conclusion [8, 14, 16, 17, 35, 38–41], where there is no significant difference between sexes. However, contradictory results have been reported by Hsiao et al. [42] and Zhang et al. [43], where the DTI-ALPS index in women was larger than in men. This shows that sex differences might still need further investigation into different illnesses with a larger sample size.

Furthermore, we did not see a significant difference between the DTI-ALPS index and the type of tumor (primary or brain metastasis). In contrast to Toh et al. [11], we did not find a linear correlation between the tumor ADC and DTI-ALPS index. However, due to our small sample size, further studies are needed to evaluate these parameters. These contradicting results could be due to differences in calculating the ADC in the tumor. We selected a small area in the middle of the tumor, while they calculated the ADC by making an average of all slices where the tumor was present. However, we did not investigate PTBE and thus cannot directly compare this parameter.

Previous studies found a negative correlation between age and DTI-ALPS index [5, 15, 25–27, 42, 44, 45]. This is a common finding in healthy volunteers and in neurodegenerative diseases such as Parkinson’s and Alzheimer’s disease. However, in cases of brain tumors [9–11], REM sleep disorder [46], neuromyelitis optica [47], renal disease [48, 49], global amnesia [50], and migraine [36] did not find significant correlation was found between DTI-ALPS and age. In our research, we did also not find any correlation between patients' age and DTI-ALPS index. Therefore, the relationship between age and DTI-ALPS index should be further studied to determine which factors might be involved in the diffusion changes along perivascular spaces under other clinical conditions.

### Study limitations

Here, we compare the DTI-ALPS index of the brain tumor hemisphere with the contralateral side and also differences between tumor type, age, and sex. Additionally, we included HC to compare the DTI-ALPS indexes. Nonetheless, there are several limitations that need to be addressed:

The sample size of this single-center, retrospective, consecutive study is small. Further studies should consist of more tumor patients and age- and sex-matched HC. Additionally, the study lacks data about the clinical course of the patients; thus, we cannot correlate GS dysfunction with subsequent, distinct psychiatric or neurological deficits. Further studies should therefore correlate the DTI-ALPS index with corresponding neuropsychological parameters.

The correct planning of the ROIs can be difficult and may require practice. In this study, a senior and junior neuroradiologist positioned the ROIs in consensus. Additionally, we planned the ROIs using only the fractional anisotropy image, as susceptibility-weighted images are not part of the clinical routine at our institution. Further studies should include susceptibility-weighted images for positioning of the ROIs to determine whether the course of the periventricular and peritumoral blood vessels has an effect here.

Additionally, the DTI-ALPS method makes an estimation of the diffusivity along the perivascular space in a small ROI parallel to the medullary veins. Therefore, makes an extrapolation that the whole brain glymphatic function could be derived from this small area within deep white matter, while the production and accumulation of amyloid β and tau proteins occurs in the brain cortex [51].

It is important to consider that the DTI-ALPS index does not solely reflect random water motion in the perivascular spaces, but it can be also influenced by factors such as axonal degeneration. Therefore, even if the DTI-ALPS appears as a technique to evaluate the GS, those indices might not reflect brain waste clearance directly. Hence, changes in the DTI-ALPS index should be interpreted with caution [37, 52, 53].

## Conclusion

The DTI-ALPS appears to be an excellent noninvasive technique to evaluate GS function. The lower DTI-ALPS index of the ipsilateral hemisphere might suggest that GS function is impaired in patients with brain tumors. Although the DTI-ALPS index was larger on the contralateral side, one should not consider this value as a healthy control. As brain tumors often affect larger areas, if not the whole brain, one could expect a general GS disruption. As shown here, the contralateral side in patients had a larger DTI-ALPS index than HC, maybe to increase the metabolic waste clearance. However, no definitive conclusions can be drawn yet. Further studies are needed to investigate the GS and its relationship between the DTI-ALPS index and different histological brain tumor entities. The DTI-ALPS index might be suitable as an initial biomarker for GS status and function in patients with tumors in the brain. However, as the factors influencing the DTI-ALPS index are still unknown, the interpretation of changes in the index should not be directly linked to GS function. Caution in drawing conclusions is advised.
